# Adult zebrafish as advanced models of human disease

**DOI:** 10.1242/dmm.050351

**Published:** 2023-07-31

**Authors:** Richard M. White, E. Elizabeth Patton

**Affiliations:** ^1^ Ludwig Cancer Institute, Nuffield Department of Medicine, Old Road Campus Research Building, University of Oxford, Roosevelt Drive, Oxford OX3 7DQ, UK; ^2^ MRC Human Genetics Unit, CRUK Scotland Centre and Edinburgh Cancer Research, Institute of Genetics and Cancer, The University of Edinburgh, Western General Hospital, Crewe Road South, Edinburgh EH42XU, UK

**Keywords:** Adult zebrafish, Disease modelling, Human disease

## Abstract

Modelling adult diseases to understand their aetiology and progression, and to develop new therapies, is a major challenge for medical biology. We are excited by new efforts in the zebrafish community to develop models of adult diseases that range from cancer to heart, infectious and age-related diseases, and those that relate to toxicology and complex social behaviours. Here, we discuss some of the advances in the field of zebrafish models of adult disease, and where we see opportunities and challenges ahead.

## Teaser

**Summary:** Zebrafish embryos and larvae have greatly contributed to our understanding of human development and disease, but recent advances are now enabling the use of adult zebrafish that have unique advantages for modelling adult disease.

## The evolution of zebrafish from development to disease

Zebrafish are a small freshwater aquatic species native to South Asia. Historically, they were used as a model to test the carcinogenic properties of ethyl carbamate (also known as urethane) by examining early development, facilitated by the transparency and *ex-vivo* development of the organism (Battle and Hisaoka, 1952). By the 1960s, it was recognized that zebrafish readily developed cancer when exposed to mutagens (Stanton, 1965) and numerous studies explored a wide variety of chemicals that affected tissues of the fish. The field took a leap forward in the 1980s, led in part by the work of George Streisinger and others who recognized that zebrafish could be a useful model for genetic screens (Walker and Streisinger, 1983), paving the way to large-scale phenotypic screens in embryogenesis. This culminated in the 1996 landmark ‘Zebrafish Issue’ in our sister journal *Development*, in which mutants in nearly every organ and tissue were described (see also Mullins et al., 2021 and https://journals.biologists.com/dev/collection/6980/The-zebrafish-issue-25-years-on). This large collection of genetic mutants, still used to this day, was crucial to understand fundamental mechanisms of vertebrate development as well as human Mendelian genetic disease.

**Figure:**
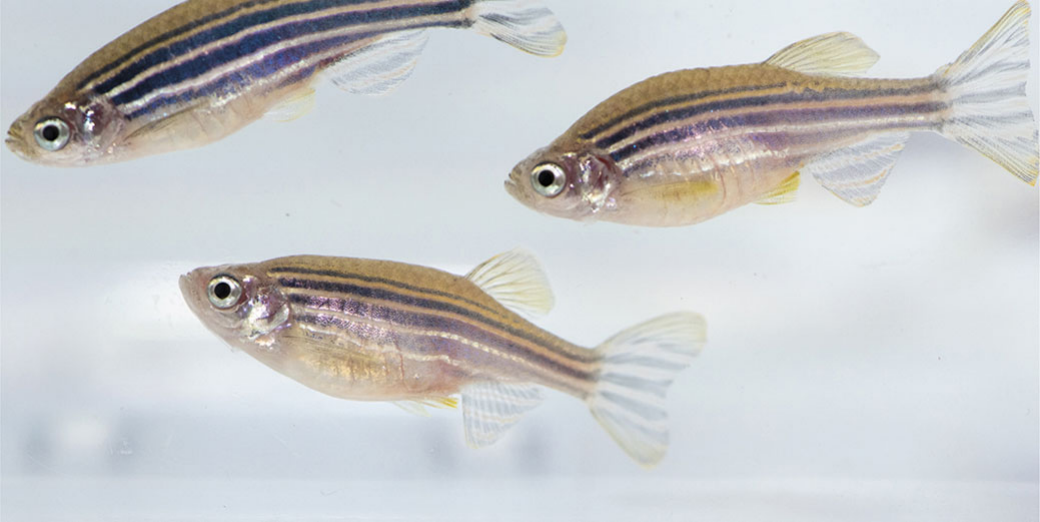
Adult zebrafish. Image courtesy of Craig Nicol, Institute of Genetics and Cancer, The University of Edinburgh. For permission to reuse, contact Craig Nicol at c.nicol@ed.ac.uk.

## What are the challenges (and solutions) for studying adult diseases in zebrafish?

Zebrafish are now employed to model a wide array of pathologies (Patton and Tobin, 2019). Despite these advances, the current methodologies are still not optimal for modelling many adult genetic diseases, especially non-Mendelian disorders. This is primarily because many studies have induced transgene expression from the one-cell stage. This does not reflect the situation in humans, in whom alterations in genes (whether by DNA mutation or by altered RNA/protein expression) can become dysregulated during adulthood. This is a key point, since altering gene function in embryonic or larval cells can have vastly different outcomes compared with altering that same gene during an adult stage or in an aging cell. On top of this, environmental influences that we experience during adulthood, such as diet, infection and pollutants, all can strongly modify the impact of any given genetic alteration.

### Inducing cell- or tissue-specific genetic alterations in adult zebrafish

Initial studies in zebrafish examined germline alleles that caused strong phenotypic defects in early development, yet, many human diseases manifest in adulthood. Therefore, a main hurdle that persists is the relative lack of models that allow the induction of genetic alterations at later life stages or the tracking of individual cells within a defined period of adult disease progression. The advent of transgenesis in zebrafish has enabled cell type-specific gene editing in embryos by using a promoter/enhancer specific to a given cell type or cDNA cassettes only in the tissue of interest, which allows the dissection of cell-autonomous and non-cell autonomous functions in disease phenotypes (Isiaku et al., 2021). However, these advanced technologies often remain out of reach for adult models. Generating somatic mutations in zebrafish adult models has been facilitated by recent work in our lab (R.M.W.), using Transgene Electroporation in Adult Zebrafish (TEAZ) (Callahan et al., 2018). With this method, specific transgenes or gene editing constructs can be directly electroporated into the adult zebrafish skin, generating somatic mutations in a spatially and temporally controlled manner. This has been a powerful method to develop new genetic models of adult zebrafish melanoma (Baggiolini et al., 2021) and metastasis (Suresh et al., 2023), but this technique has limitations when targeting tissue that is less accessible.

Inducible models for fate mapping are widespread in the zebrafish embryo, flies and adult mice, but applying these to adult zebrafish has been often hampered by difficulties in optimisation for the adult stages and problems with toxicity, issues that are not always easy to troubleshoot within the parameters of an animal experiment license. One elegant example of lineage tracing in adult zebrafish from the Laure Bally-Cuif’s laboratory reports the fate mapping of adult neural stem cells in the zebrafish brain (Than-Trong et al., 2020). In our own lab (E.E.P.), tamoxifen-inducible cre^ERt2^/loxP lineage tracing in adult zebrafish was applied to follow individual ‘persister cells’ in residual melanoma disease, which proved that these cells undergo transcriptional plasticity or ‘cell state switching’ to contribute to recurrent disease (Travnickova et al., 2022). As we now aim to apply this technique more widely, we are finding that – similar to when using mouse models (Jahn et al., 2018) – each cre^ERt2^ promoter requires its own specific tamoxifen-optimization protocols. These protocols will be important to share openly within the zebrafish community to accelerate disease modelling, and to limit repetitive and unnecessary animal experiments. As inducible Cre techniques improve, we will be able to incorporate innovative Cre-enabled transgenes in adults, such as the recently described Cre-enabled tetracycline-inducible transgenic system for tissue-specific cytokine expression (CETI-PIC3) that enables a controlled method to study diseases with an underlying inflammatory aetiology (Ibrahim et al., 2020).

Another potential application for this is in the investigation of how tumour cells interact with cells in the tumour microenvironment (TME), such as T cells, B cells and fibroblasts. These interactions are not static, as the cells reprogram each other through exchange of metabolites, signalling molecules and extracellular vesicles. For these reasons, it is important that we develop techniques to knock out or overexpress genes in each relevant cell type in an inducible manner, and then enable imaging of each ‘partner’ in the interaction between tumour and TME. Ultimately, this will allow us to analyse how cells interact with each other over time and will help to understand the mechanism, and cellular dynamics behind the progression of adult disease.

Emerging gene editing techniques could also have immense potential if adapted for use in adult zebrafish. First-generation (F0) mosaic mutant zebrafish generated by CRISPR/Cas9-directed genome editing (also known as crispant zebrafish) induce non-germline editing with increasing efficiency by using recombinant Cas9 protein incubated with a synthetic stabilized single guide RNA (sgRNA) (Burger et al., 2016; Kroll et al., 2021). This general idea can also be used in plasmid-based systems, such as modelling approach in zebrafish for rapid tumour initiation (MAZERATI), in which mosaic expression of sgRNAs can be coupled with expression of a tissue-specific Cas9 (Ablain et al., 2015). However, currently, neither crispants nor MAZERATI are inducible, so adaptations to allow the introduction of these systems in a latent form, to be activated at later stages would be useful. A similar argument could be made for Cas13 subtype (Cas13d) enzymes, which modify mRNA and have demonstrated high efficiency at knocking genes down (Kushawah et al., 2020). A relatively less-explored area is the use of protein degradation systems, known as degrons, in which a protein of interest can be rapidly degraded after drug application. Early use of these technologies in zebrafish (Yamaguchi et al., 2019) and *C. elegans* (Hermann et al., 2015) has shown great promise. Coupling these methods with alternative strategies, such as electroporation, is another approach to enable tissue- and time-specific perturbation of gene function. Optimization of inducible technologies combined with non-germline approaches will allow us to harness the true utility of the fish to study multifactorial diseases.

#### Gene–environment interactions in adult zebrafish

Many common adult diseases in humans, such as cancer and heart disease, involve multifactorial combinations of genetic variants with environmental factors and lifestyle. The adult zebrafish is an exciting model to reveal fundamental principles of gene–environment interactions, exemplified in a recent DMM paper from our lab (R.M.W.) in which we explore the interactions between diet, sex and genetics in melanoma (Montal et al., 2023). In human populations, complex heart diseases are also often affected by ageing. Bill Chaudhry and colleagues tested the effects of swimming exercise and ageing on the adult zebrafish heart and found that, while prolonged exercise can improve the fitness in ageing animals, it can also increase fibrosis in the heart (Murphy et al., 2021). Incorporating some of the new phenotyping tools for electrocardiogram data in adult zebrafish will be especially interesting to apply to ageing animals that undergo different exercise regimes (Duong et al., 2021)**.**

As the human immune system changes significantly throughout life – which affects responses to infection or toxic substances – it is important to study infection and immune-related diseases in adult model organisms. One bacterial infection that has been extensively studied in zebrafish, including adults, is *Mycobacterium marinum*, a close genetic relative of the human pathogen *Mycobacterium tuberculosis*. Using adult zebrafish in infection studies has helped build our understanding of the role of adaptive immune responses to mycobacterial infection (Swaim et al., 2006) and could lead to the development of improved vaccines for tuberculosis (Saralahti et al., 2020). Furthermore, introducing mutations or knockout of key immune signalling molecules to adult zebrafish has dissected the complexity of immune responses to *M. marinum* infection (Brewer et al., 2022), including more-unique features, such as granuloma formation (Cronan et al., 2021), that are conserved in tuberculosis in humans (Ramakrishnan, 2023).

The majority of adult diseases involve complex interplays of germline genetics, somatic alterations (DNA, RNA, protein) and environmental exposures. Zebrafish are ideally suited for examining this interplay because, compared with mammalian models, they are much easier to manipulate in large numbers. For example, fish can be engineered to overexpress a gene of interest in a specific cell type, in a specific genetic background, and then fed different diets or exposed to varied stimuli and examined at different ages. To study each of these factors *in vivo* requires a large sample size that can test them in isolation as well as in a combinatorial fashion, something that is uniquely achieved in zebrafish. However, these sample sizes are further expanded when also considering gene–gene interactions. For instance, there can be significant phenotypic variation between patients comprising the same causative genetic mutation but different modifier gene mutations. Screens for potential modifier genes that contribute to phenotypic variation can create huge demand on facilities and staff as they handle multiple generations of large numbers of double mutant animals. To circumvent this issue, Xiaolei Xu and colleagues adapted an F0-based CRISPR screen that uses microhomology-mediated end-joining (MMEJ)-based genome technology to enable precise and homogenous gene editing that persists into adulthood, and allows swift identification of modifier genes in a zebrafish model of dilated cardiomyopathy (Ding et al., 2022). Advances like this can further potentiate the use of adult zebrafish in interrogating complex disease aetiology.

#### Staying transparent in adult zebrafish

Live imaging of single cells and tissues over time remains one of the most exciting aspects of disease modelling in zebrafish, and yet this becomes significantly more complex in adult zebrafish. To begin with, zebrafish embryos are transparent while adult zebrafish lose this transparency and are heavily pigmented. Genetic crosses of a mutation in the pigmentation gene *mitfa* in melanocytes and of a mutation in *roy* (also known as *mpv17*) in shiny iridophore cells, have generated the widely used transparent zebrafish ‘*casper*’ (White et al., 2008), which facilitates imaging in both larvae and adults. Furthermore, handling adult fish is more complex than zebrafish embryos, as the latter can survive embedded in agarose with anaesthetic for days. To address this issue, Brant M. Weinstein’s laboratory has improved protocols for extended imaging of adult zebrafish by using intubation that permits over 20 h of live imaging to capture neutrophils responding to a wound (Castranova et al., 2022). By combining the benefits of both transparency and small organism size, with the complexity of adult brain biology, Pui-Ying Lam has developed methodology for prolonged, longitudinal imaging of the small and life-long optically transparent *Danio* species *Danionella cerebrum* (Lam, 2022). Access to live imaging of the adult zebrafish brain often requires surgery but *Danionella cerebrum* lacks dorsal skull bones, enabling direct access for confocal imaging. This way, Lam was able to image the dynamic cellular processes of highly motile macrophages and microglia cells in response to brain injury.

### Drug development in adult zebrafish

Drug development in zebrafish has directly contributed to new drug treatments that are in the clinic or in clinical trials (Baraban, 2021; Patton et al., 2021). Zebrafish are amenable to high-throughput drug screens because of their small size and ability to absorb drugs directly from the water. However, as adult zebrafish are larger and less amenable to high-throughput analysis than embryos or larvae, more technical specialization is required. In particular, drug administration is harder in adult zebrafish: drugs can be added to the water and absorbed through the skin and gills, but this results in variable absorption rates, toxicity and is only useful for water-soluble compounds. To address this, Leonard Zon’s laboratory pioneered oral gavage methods that allows for dose-controlled administration of compounds (Dang et al., 2016). Further, our laboratory (E.E.P.) has recently developed a drug pellet method through which dose-controlled BRAF inhibitors – which are hydrophobic – can be administered without the need for animal handling, demonstrating a highly specific and non-toxic inhibition of MAPK signalling within the tumour (Lu and Patton, 2022).

Drug testing in the adult zebrafish offer a new opportunity to understand how drugs function, and how they are metabolized within a whole animal and in specific organs and tissues. Gary Patti and Leah Shriver’s laboratories have recently developed a multidimensional metabolomics platform to investigate the mode of action and toxicity of drugs in adult zebrafish by combining an array of techniques, including mass spectrometry and *in vivo* isotope tracing (Jackstadt et al., 2022). In this context, there are two exciting advantages of the adult zebrafish: individual adult zebrafish organs can be dissected and evaluated to directly assess drug biodistribution and pharmacodynamics. Moreover, due to the size of an adult zebrafish, a cross-section of the entire animal can be analysed by mass spectrometry imaging (MSI) to simultaneously visualise drug metabolism in different organs.

Patient-derived xenograft (PDX) models are commonly generated in mice to test cancer drug options and predict individual patient responses. Zebrafish PDX (zPDX) models, known as avatars, have immense benefits as they are faster and cheaper to generate than mammalian models and, due to their relatively small size as adults, offer more high-throughput capabilities compared to mice. Recent advances in adult zPDX models have been made, as David Langenau’s laboratory generated stably immunocompromised adult zebrafish through genetic ablation of T, B and natural killer (NK) cells (Yan et al., 2019), meaning that the tumours are not rejected. These zebrafish displayed prolonged and robust engraftment of several human cancer cell lines with similar efficiency to mouse PDX models, and were used to examine potential therapies for rhabdomyosarcoma, which has led to new clinical trials (www.clinicaltrials.gov, NCT01858168). Therefore, adult zebrafish can act as a preclinical model to support precision cancer medicine.

#### Subtlety in phenotyping adult disease

Many of the current phenotyping assays employed in zebrafish models of disease are recorded through binary classifiers, establishing, for example, whether the fish has cancer or not. But for many adult diseases, phenotypes in patients are much more subtle and graded. A good example of this are neuropsychiatric conditions like anxiety, addiction and sleep disorders. These disorders can be effectively modelled in zebrafish owing to a high level of evolutionary conservation in the subcortical social brain between teleosts and mammals (Geng et al., 2023). Furthermore, there are increasing numbers of assays to evaluate the complex behaviours associated with these conditions in adult zebrafish, as reviewed by Geng et al. (2023). These include assays to examine anxiety-like behaviours that, in one example, revealed the impact of antibiotic exposure on adult zebrafish behaviour (Gusso et al., 2021). Randall Peterson’s lab recently tracked various behaviours in an adult zebrafish model of a neurodegenerative lysosomal storage disorder to identify a potential therapeutic target and assess the efficacy of pharmacological or genetic rescue (Zhang et al., 2023). Excitingly, a phenomics platform called ZeChat has been developed to examine factors, such as sociality, via an unsupervised deep learning method (Geng et al., 2023), a good example of finding ever more complex phenotypes by using zebrafish screens.

## Where do we go from here?

The evolution of zebrafish as a disease model has been remarkable to witness, bringing classic genetic tools to new areas of biology. While we are excited by the advances and potential for zebrafish as a model of adult disease, it is sometimes difficult to effectively communicate the success of our model to a wider audience. We are sensitive that research on animals is – both from the political side and in the eyes of the public – under scrutiny. One issue, also recently highlighted in the Science article ‘*Germany weighs whether culling excess lab animals is a crime*’ by Feldwisch-Drentrup (2022), is the on-going discussion in Germany into the legality of culling surplus animals. This is especially problematic for researchers involved in the development and maintenance of mutant animal lines. Furthermore, there is the current ‘End Animal Testing’ European Citizens’ Initiative (https://www.endanimaltesting.eu/) that, at the level of the European parliament, effectively aims to end all experimental animal work. Although the parliamentary outcome of these campaigns may be a strengthening of the Replacement, Reduction and Refinement (3Rs) principles (https://www.nc3rs.org.uk) rather than a ban of animal experimentation, it is evident that a more-open public discussion around animal experimentation is needed.

Public perception of experimentation in zebrafish is often not as negative as it is for mammalian laboratory models, but the zebrafish community can support its own cause by optimising and standardising husbandry and breeding protocols. First, we should ensure that genetic lines and protocols are freely shared, and that freezing protocols for sperm are optimised to reduce the breeding of zebrafish simply to maintain genetic lines. The newer mosaic strategies described above (Ablain et al., 2015; Burger et al., 2016) are powerful but cannot fully replace well-characterised genetic mutant lines. However, they may provide an inroad to help reduce surplus animals that would otherwise be generated through genetic crosses. Second, the zebrafish research societies should continue to foster and intensify open dialog with the veterinary and regulatory bodies about zebrafish science and welfare. In this regard, the recent collaboration between the European Zebrafish Society and the Federation of European Laboratory Animal Science Associations has produced a series of guidelines that act to both standardise zebrafish husbandry protocols for experimental reproducibility across laboratories, and provide guidance on best practices for zebrafish husbandry and welfare (Aleström et al., 2020). Another area that requires a coordinated effort between the various zebrafish societies and government authorities, is the question of whether zebrafish can be used as the basis for FDA- or EMA-approved trials, especially when novel compounds have been identified. Working together to directly face these barriers and to identify opportunities, we can maximise the use of zebrafish as physiologically meaningful models of human disease.
